# A Web Server for GPCR-GPCR Interaction Pair Prediction

**DOI:** 10.3389/fendo.2022.825195

**Published:** 2022-03-24

**Authors:** Wataru Nemoto, Yoshihiro Yamanishi, Vachiranee Limviphuvadh, Shunsuke Fujishiro, Sakie Shimamura, Aoi Fukushima, Hiroyuki Toh

**Affiliations:** ^1^ Division of Life Science, Department of Science and Engineering, School of Science and Engineering, Tokyo Denki University (TDU), Hatoyama-machi, Japan; ^2^ Master’s Programs of Life Science and Engineering, Graduate School of Science and Engineering, Tokyo Denki University (TDU), Hatoyama-machi, Japan; ^3^ Department of Bioscience and Bioinformatics, Faculty of Computer Science and Systems Engineering, Kyushu Institute of Technology, Iizuka-shi, Japan; ^4^ Bioinformatics Institute (BII), Agency for Science, Technology and Research (A*STAR), Singapore, Singapore; ^5^ Department of Biomedical Chemistry, School of Science and Technology, Kwansei Gakuin University, Sanda-shi, Japan

**Keywords:** GPCR, protein-protein interaction, membrane protein, disease-associated mutation, machine learning, web service, prediction, bioinformatics

## Abstract

The GGIP web server (https://protein.b.dendai.ac.jp/GGIP/) provides a web application for GPCR-GPCR interaction pair prediction by a support vector machine. The server accepts two sequences in the FASTA format. It responds with a prediction that the input GPCR sequence pair either interacts or not. GPCRs predicted to interact with the monomers constituting the pair are also shown when query sequences are human GPCRs. The server is simple to use. A pair of amino acid sequences in the FASTA format is pasted into the text area, a PDB ID for a template structure is selected, and then the ‘Execute’ button is clicked. The server quickly responds with a prediction result. The major advantage of this server is that it employs the GGIP software, which is presently the only method for predicting GPCR-interaction pairs. Our web server is freely available with no login requirement. In this article, we introduce some application examples of GGIP for disease-associated mutation analysis.

## Introduction

G Protein-Coupled Receptors (GPCRs) form higher-order molecular complexes (oligomers) with other GPCRs. The molecular functions of such oligomers differ from those of monomers with respect to at least one of the following examples: endogenous ligand binding, coupling with trimeric G-proteins, expression levels on membrane, and intracellular trafficking. The regulatory chemicals of oligomer formation are likely to work by different mechanisms from those of the existing GPCR-targeted chemicals (1–6). GPCR-GPCR interactions are unique in that GPCRs with different molecular functions interact with each other and exert molecular functions that are completely different from those of the monomers, as reviewed previously (7–9). GPCR hetero-dimers are considered to be novel therapeutic targets (10).

There are various methods to predict interacting pairs between soluble proteins (11) or between soluble and membrane proteins (12). These methods cannot be applied to predict interacting GPCR pairs, since the physicochemical properties at the interfaces between soluble proteins or between soluble and membrane proteins are different from those between membrane proteins. Unlike globular proteins, membrane proteins reside within the lipid-bilayer that surrounds cells and organelles, and their exposed residues are more hydrophobic than the buried ones. The native structures of membrane proteins collapse when removed from their natural membrane environment, because they require interactions with the lipid environment for their structural stability. This is reflected in the fact that transmembrane proteins, as listed in the latest version of PDBTM (13), represent only 6,757 out of a total of 196,672 protein-only structures in the PDB; that is, ∼3.4%. As described in the article about PPIMem (14), consequently, the molecular complexes formed between membrane proteins are represented by an even lower ratio, about 1.4% (619/44,700), for protein–protein non-covalent dimer complexes as defined by the PDBePISA v1.52 server (https://www.ebi.ac.uk/msd-srv/prot_int/pistart.html). These technical problems have delayed comprehensive investigations of membrane proteins and their functions associated with their higher-order structures (15).

The recent release of state-of-the-art methods, AlphaFold2 (AF2) (16) and RoseTTAFold (RF) (17), might change the situation. AF2 and RF are capable of predicting the 3D structures of input sequences with extremely high accuracy. Since the release of the source codes of both tools, discussions about their application limits and applicability have continued (18–25). These software tools are considered to predict homo- or hetero-complex structures as well as monomer structures, although researchers around the world are currently testing whether AF2 can predict complex structures with the same accuracy as monomeric structures. The execution of the original versions of AF2 and RF requires a large amount of computing resources, and the users’ command line skills. Without such skills, Colabfold (23), which outputs homo- or hetero-complex structures by setting the “model_type” option to AlphaFold2-multimer and using the sequence pair as a query, can be employed. Using this option, we may be able to predict the structures of GPCR heterodimers.

Colabfold is an easy-to-use Notebook-based environment for fast and convenient protein structure predictions (23). Its structure prediction is powered by AF2 and RF, combined with a fast multiple sequence alignment generation using MMseqs2 (26). Colabfold generates multiple models with the average pLDDT and the pTMscore. When we modeled human rhodopsin homo-dimer by using Colabfold by setting the “model_type” option to AlphaFold2-multimer, the conformations of the proposed structures were completely different from each other. One of them seemed to be modeled based on the artificial crystal complex structure as the template, such as the bovine rhodopsin structure (PDB ID: 1L9H), where the topology of one protomer against the membrane plane was anti-parallel to that of the other protomer. In other words, the region of one chain that should be on the extracellular side was located on the intracellular side. This problem might be serious, because the results are the same with or without the use_templates option, which is one of the parameters used to run the prediction. This is probably due to the fact that AF2 learns the features of sequence and structure of artificial complex states, such as 1l9h.pdb. The user needs to determine which is the correct complex structure by taking the scores into account. Colabfold outputs a complex structure, but whether its constituents actually interact has not yet been verified. In particular, as far as we tried, Colabfold suggested complex structures for non-interaction pairs. Then, the pLDDT values along the interface residues were high. Thus, it may be difficult to distinguish between interaction and non-interaction pairs using only Colabfold. Creating a model structure of a complex should be treated as a separate issue from predicting whether or not a complex will be formed. As a preliminary step to the complex structure modeling, prediction of interaction pairs is helpful in research.

We previously developed GPCR–GPCR interacting pair predictor (GGIP), a method to predict specifically interacting pairs for GPCR hetero-oligomerization, by integrating the structure and sequence information (27). In the GGIP, different structural regions are assumed to be used for the interaction interfaces among various GPCRs, because the interfaces for GPCR–GPCR interactions are not always conserved even among closely related GPCRs. The prediction by GGIP is accomplished by the support vector machine (SVM), which classifies a given hetero GPCR pair into either an interacting pair or a non-interacting pair by using a pairwise kernel. The performance of our method was evaluated by the Receiver Operating Characteristic (ROC) curve, and the corresponding area under the curve was 0.938. To our knowledge, this is the only prediction method for interacting pairs among GPCRs (27).

GGIP classifies a given GPCR pair into either an interaction pair or a non-interaction pair by using a pair-wise kernel (27). In contrast to the state-of-the-art tools described above, there is no ambiguity in the interpretation of the prediction results. Users do not need to interpret the results although at present, GGIP does not propose complex 3D structures. Moreover, the execution time of GGIP is much shorter than that of Colabfold.

In many experiments, the molecular function analysis of GPCRs has been performed using cells overexpressing the GPCR of interest (7–9), but the existence of other membrane proteins and their interactions with GPCRs have not been taken into account. Most of the molecular functions of GPCRs reported so far have been considered as functions of the GPCR monomers of interest. Predicting the interaction pairs and considering the molecular functions of individual protomers may lead to a better understanding of their functions as interaction pairs. We expect that this will contribute to predictions of the functions of homo- and hetero-oligomers of GPCRs, which are another unsolved problem concerning GPCRs.

We have now launched a Web service to provide the predicted interfaces for GPCR oligomerization by GGIP, and have made some modifications of GGIP. This service, named GPCR-GPCR interaction pair predictor (GGIP), is freely available and there is no login requirement. GGIP could accelerate the analyses of the interactions among GPCRs, and thus contribute to the elucidation of the global structures of GPCR networks in subcellular organelle membranes as well as cell membranes (28). In addition, prediction of interaction GPCR pairs is helpful in research as a preliminary step to the GPCR complex structure modeling.

## Materials and Methods

### Implemented Method

GGIP is based on an SVM that is applied to the analyses of biological problems (29–35). By learning a set of positively and negatively labeled training samples, the SVM classifies new unlabeled test samples. In this study, we regarded the pair of the *x* and *y* GPCRs as the sample, and used the SVM to classify GPCR-GPCR pairs into either the interaction or non-interaction class.

**Figure 1:**
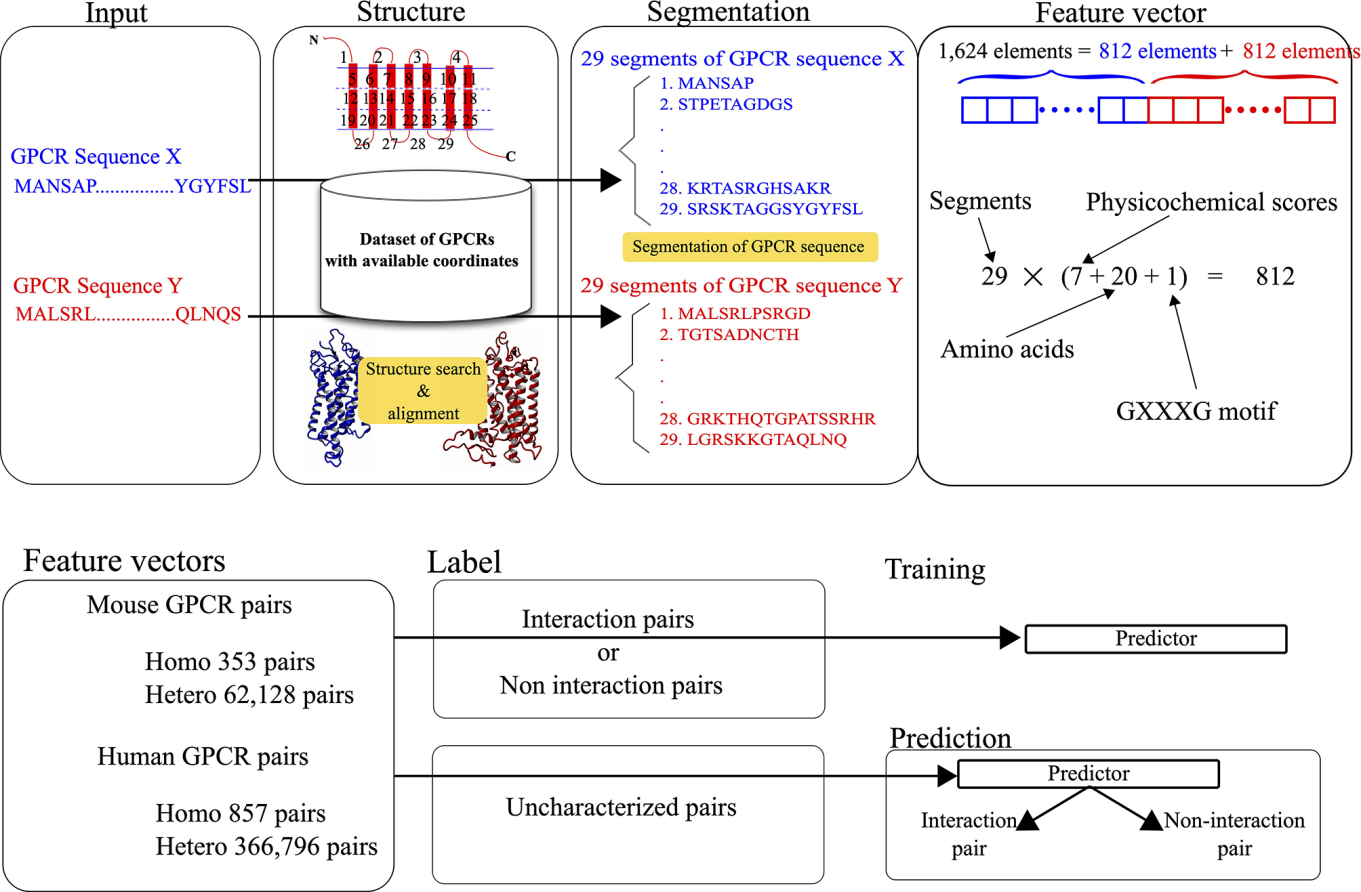
Data processing for the development and prediction of GGIP.

The feature vectors for the pairs were generated by integrating the structure and sequence information. The structural and physicochemical properties of each amino acid constituting a query sequence were evaluated and used as elements of each feature vector. As shown in **Figure 1**, the feature vector for each pair consists of 1,624 elements, where two sets of 812 scores corresponding to target GPCRs are integrated. The 812 scores consist of 28 scores for the defined 29 segments of each GPCR. To represent the features, 28 scores are calculated for each segment. The calculation procedure for the scores is identical to the one in our previous work (27).

The latest version of GGIP implemented on our server is updated from the version reported previously (27). First, the latest version uses human sequences as well as mouse sequences as training samples, while the previous version used only mouse sequences. Second, heterodimerized pairs as well as homodimerized pairs are used for positively labeled samples. Details about the learning procedure are described in the next section.

### Preparation of Training Data and Prediction Targets

In this study, we used 353 mouse sequences and 857 human sequences for training and prediction. The procedure to prepare the training data and prediction targets is the same as that described in the previous work. The procedure is briefly summarized below.

We used 353 mouse non-odorant GPCR sequences with available gene expression profiles in 41 tissues, and 857 human sequences. These target amino acid sequences were retrieved from the RefSeq database. The numbers of all possible homo- and hetero-pairs of the 353 mouse GPCRs and 857 human GPCRs are 62,481 (353 + _353_C_2_) and 367,653 (857 + _857_C_2_), respectively. Pairs between mouse and human sequences are not considered. The total number of all possible pairs is 430,134 (62,481 + 367,653).

Among them, 61 mouse hetero-pairs that were used in our previous work, and their 45 human orthologous pairs, were treated as positive data for training to develop the predictor. Among the mouse 61 pairs, 16 pairs were mouse non-odorant GPCR pairs in the GPCR oligomer databases (36–38). Referring to the databases and the literature, the remaining 45 pairs were identified in non-mouse species, but their orthologous pairs were present in mice. Orthologous relationship was identified by referring to HomoloGene database, which contains automatically generated sets of homologous genes and their corresponding mRNA, genomic, and protein sequence data from selected eukaryotic organisms (39). All the homo-pairs were regarded as positive data because there are no data reported for GPCRs that do not form homodimers, and as described by Borroto-Escuela et al. (38), more than 87% of the total identified protomers exist as homomers.

In addition to the 9,275 negative data candidates previously used for the GGIP development, their human orthologous pairs were also included among the candidates. The interactions of the remaining 410,252 pairs have not been characterized yet, and thus can be used to predict novel interacting pairs.

The negative pairs for the training were randomly selected from the negative data candidates. The size of the selected negative data set was set to three times greater than the number of positive pairs. As described in our previous work (27), this is based on the evaluation of the effects of data imbalance by varying the ratio between positive and negative samples (e.g., 1:1, 1:2, 1:3, 1:4, 1:5). Then, the experimentally suggested interacting and non-interacting pairs were always selected as the positive and negative data.

## Usage

### GGIP Input Page

**Figure 2:**
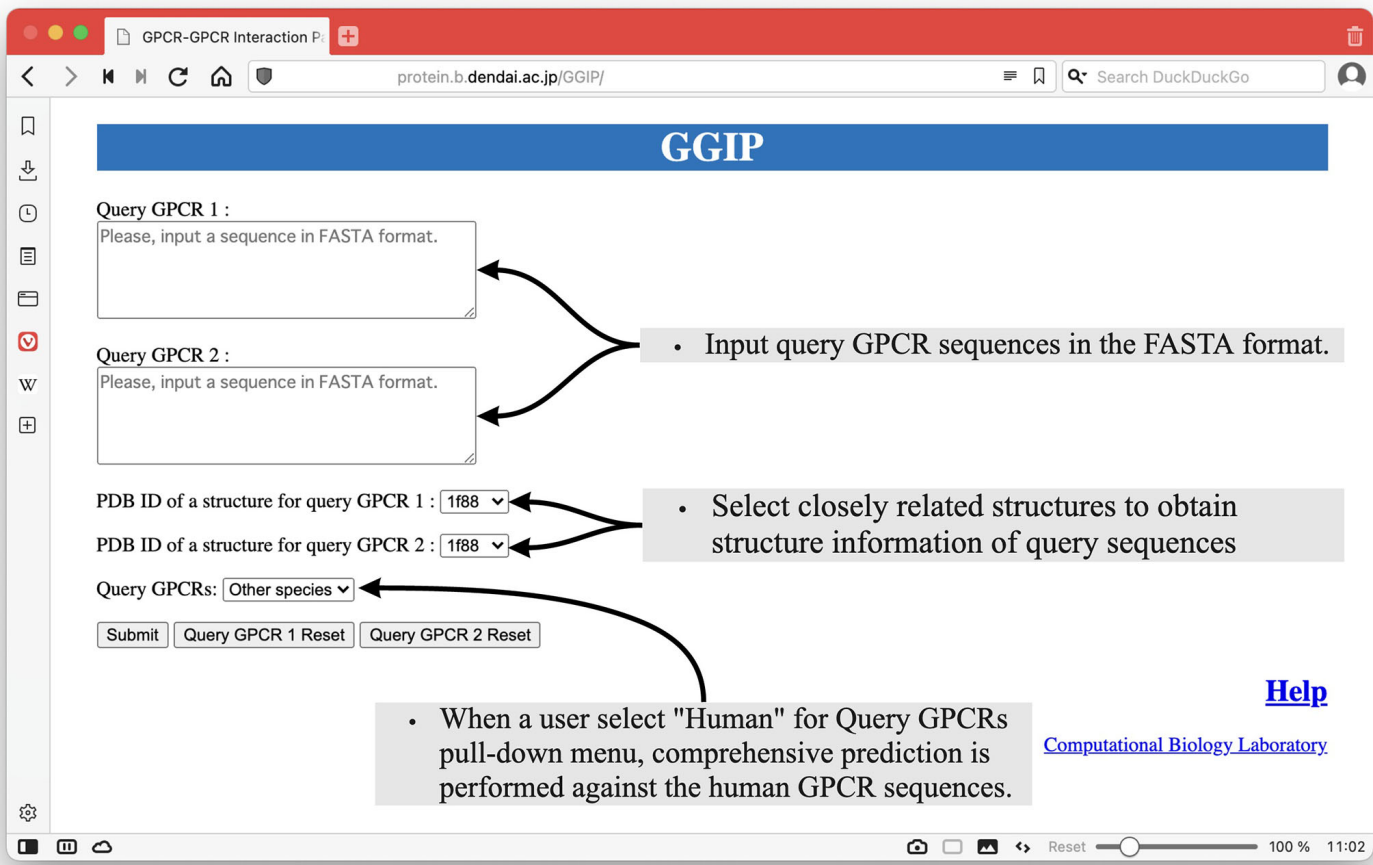
Input page of GGIP.

As shown in **Figure 2**, there are two text areas on the left side of the Input page for the input of the query pair sequences. Each amino acid sequence constituting a GPCR pair in the FASTA format is required for each textbox to execute GGIP. Each sequence is transformed into a feature vector. There are two selection boxes showing the list of the available structure data in the server. Each amino acid in each sequence is mapped to an amino acid in the selected structure. Most of the tertiary structure data specified by a single PDB ID are composed of multiple chains. The three-dimensional structural data thus provided are pre-processed in the same way as that described above.

At the bottom left of the Input page, there is a pull-down menu to confirm that the input sequences are human sequences. When this checkbox is selected, comprehensive prediction is performed against the human GPCR sequences under the assumption that the input sequences are human GPCR sequences. The predicted interaction partners of the query sequences are predicted and displayed on the results page.

### Computational Platform

The GGIP server has been confirmed to function on Vivaldi 4.1.2369.16 (Mac OSX Big Sur 11.5.2), Chrome 92.0.4515.131 (Mac OSX), Safari 14.1.2 (Mac OSX), and Chrome 91.0.4472.123 on Windows 10. There is no software requirement to execute the prediction and display the prediction results.

### Calculation Time

The calculation time is about 20s when the human rhodopsin sequence pair is used as a query, which is available as an example on the HELP page. The calculation times for the other examples provided on the page are almost the same as that for the rhodopsin sequence. These durations were measured on a 2.4 GHz AMD Ryzen 9 3950X 16-Core Processor, running Ubuntu 20.04.2 LTS.

### Result Page

**Figure 3:**
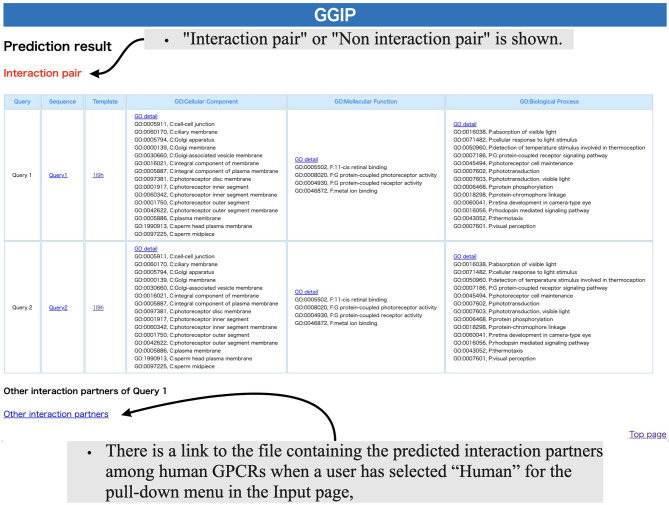
Results page of GGIP.

As shown in **Figure 3**, the Result page shows whether the query pair was predicted to interact or not. The GO terms of the input GPCRs are shown in three categories, Cellular Component, Molecular Function, and Biological Process, and the functions of the two GPCRs can be compared. If the human element is selected in the pull-down menu on the Input page, then the query pair is assumed to be human GPCRs, and an exhaustive prediction of interaction pairs with human GPCRs is performed. A link to the file with the prediction results will be displayed at the bottom of the page. The file contains the accession numbers of the predicted interaction partners of each query.

## Examples

Three examples with appropriate parameter settings and results are available in the ‘Examples’ section on the HELP page.

### Practical Examples

In this study, by using GGIP, we show examples that have investigated disease-associated mutations that may affect GPCR interactions. We have compared the predicted results when a wild-type GPCR sequence pair is used for the prediction with those when a disease-associated mutation is introduced into either query sequence. When a wild-type pair is predicted to be an interaction pair and a pair containing a mutation is predicted to be a non-interaction pair, the mutation is regarded as an interaction inhibitive mutation (IIM). In contrast, when the wild-type pair was predicted to be a non-interacting pair and a pair containing a mutation was predicted to be an interacting pair, the mutation is regarded as an interaction promotive mutation (IPM).

**Figure 4:**
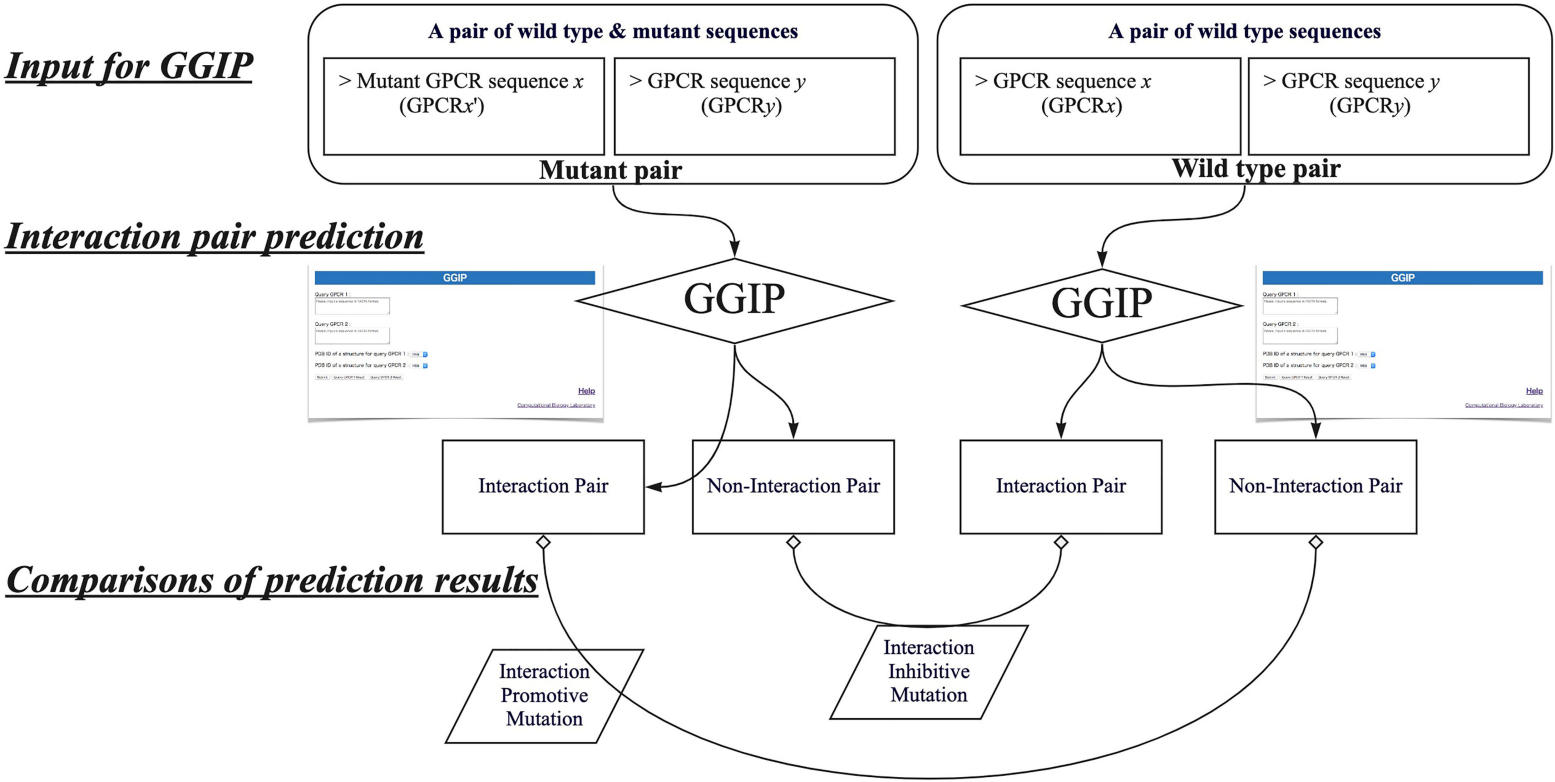
Workflow to predict interaction inhibitive mutations and interaction promotive mutations.

**Figure 4** describes the workflow to predict that a missense mutation m in GPCRx is IIM or IPM. The mutant sequence created by introducing the missense mutation m into the GPCRx sequence is designated as GPCRx’. The interaction partner of GPCRx is denoted by GPCRy. A pair of GPCRx and GPCRy is referred to as a wild type pair, and a pair of GPCRx’ and GPCRy is a mutant pair. Both pairs are used for GGIP input data. GGIP is a two-class classification method based on SVM. Both pairs are classified as either an interaction pair or a non-interaction pair, and thus there are a total of four possible combinations of prediction results. If the mutant pair and the wild-type pair were predicted to be a non-interaction pair and an interaction pair, respectively, then the mutation was considered as an IIM. If the mutant pair and the wild-type pair were predicted to be an interaction pair and a non-interaction pair, respectively, then the mutation was considered as an IPM. It should be noted that our method is not designed to predict mutations that will only strengthen or weaken the degree of interaction. Our method also does not aim to predict mutations that disrupt the conformation of the protomer but maintain the interaction. Lastly, our method is not designed to predict mutations that alter the function of the complex but maintain the interaction.

The first example is the analyses of three mutants of human rhodopsin causing retinitis pigmentosa (RP) (40). Three RP-associated single mutations, F45L (TM1), V209M (TM5) and F220C (TM5) in human rhodopsin were confirmed to behave as monomers in pull-down experiments, although wild-type rhodopsin functionally reconstituted into liposomes exists as dimers or multimers (40). It was speculated that these mutants were not able to dimerize during detergent-mediated reconstitution, and that this defect manifested itself in the dysregulation of rhodopsin organization in disc membranes, potentially affecting many aspects of disc biogenesis and maintenance, and ultimately leading to disease pathology (40). All the RP-associated mutants were predicted as IIMs by using GGIP. A pair of wild-type human rhodopsin sequences were predicted to interact, while all the three RP-associated single mutants were predicted not to interact with the wild-type rhodopsin.

The second example is the analyses of human GABA_B_R_2_ (GB2) mutations causing epileptic encephalopathy (EE) and Rett-like syndrome (RS) (41). EE-associated mutations, G693W, S695I, and I705N in human GB2, and an RS-associated mutation, A707T in human GB2, were confirmed to reduce the number of heterodimers. In our analysis, all the EE-associated and RS-associated mutants were predicted as IIMs. The wild-type human GABA_B_R_1_ (GB1) sequence and the GB2 sequence were predicted to interact with each other by our method, while the EE-associated and the RS-associated GB2 mutants were predicted not to interact with the wild-type GB1 sequence.

These examinations demonstrated that our method may identify disease-associated single amino acid mutations that affect GPCR oligomerization, although further validation is required. However, it should be noted that our method still has a limitation that some experimentally confirmed GPCR pairs are predicted as non-interaction pairs.

## Data Availability Statement

Publicly available datasets were analyzed in this study. This data can be found here: https://protein.b.dendai.ac.jp/GGIP/.

## Author Contributions

WN and HT conceived the basic idea of GGIP and designed the prediction method. YY provided the source code for machine learning. VL and WN discussed the interaction between disease-associated mutations and GPCRs. SF and SS extended GGIP. AF implemented a program to obtain information about GPCRs. All authors contributed to the article and approved the submitted version.

## Funding

Two authors (WN and HT) are supported by Grants-in-Aid for Scientific Research from the Ministry of Education, Culture, Sports, Science and Technology of Japan (25870764, 18K06199). WN is supported by ﻿the Tokyo Denki University Science Promotion Fund (Grant number: Q21L-03).

## Conflict of Interest

The authors declare that the research was conducted in the absence of any commercial or financial relationships that could be construed as a potential conflict of interest.

## Publisher’s Note

All claims expressed in this article are solely those of the authors and do not necessarily represent those of their affiliated organizations, or those of the publisher, the editors and the reviewers. Any product that may be evaluated in this article, or claim that may be made by its manufacturer, is not guaranteed or endorsed by the publisher.
