# Corrigendum: A Web Server for GPCR-GPCR Interaction Pair Prediction

**DOI:** 10.3389/fendo.2022.944910

**Published:** 2022-06-01

**Authors:** Wataru Nemoto, Yoshihiro Yamanishi, Vachiranee Limviphuvadh, Shunsuke Fujishiro, Sakie Shimamura, Aoi Fukushima, Hiroyuki Toh

**Affiliations:** ^1^ Division of Life Science, Department of Science and Engineering, School of Science and Engineering, Tokyo Denki University (TDU), Hatoyama-machi, Japan; ^2^ Master’s Programs of Life Science and Engineering, Graduate School of Science and Engineering, Tokyo Denki University (TDU), Hatoyama-machi, Japan; ^3^ Department of Bioscience and Bioinformatics, Faculty of Computer Science and Systems Engineering, Kyushu Institute of Technology, Iizuka-shi, Japan; ^4^ Bioinformatics Institute (BII), Agency for Science, Technology and Research (ASTAR), Singapore, Singapore; ^5^ Department of Biomedical Chemistry, School of Science and Technology, Kwansei Gakuin University, Sanda-shi, Japan

**Keywords:** GPCR, protein-protein interaction, membrane protein, disease-associated mutation, machine learning, web service, prediction, bioinformatics

The originally published article contained an error in the Funding statement. The grant number for the funding provided by the Tokyo Denki University Science Promotion Fund was incorrectly presented as “Q13L-03”; the correct grant number is “Q21L-03”.

The authors apologize for this error and state that this does not change the scientific conclusions of the article in any way. The original article has been updated.

## Publisher’s Note

All claims expressed in this article are solely those of the authors and do not necessarily represent those of their affiliated organizations, or those of the publisher, the editors and the reviewers. Any product that may be evaluated in this article, or claim that may be made by its manufacturer, is not guaranteed or endorsed by the publisher.

